# Population-based HIV prevalence, stigma and HIV risk among trans women in Nepal

**DOI:** 10.1186/s12879-021-05803-7

**Published:** 2021-01-29

**Authors:** Erin C. Wilson, Manisha Dhakal, Sanjay Sharma, Anuj Rai, Rajesh Lama, Sirish Chettri, Caitlin M. Turner, Hui Xie, Sean Arayasirikul, Jess Lin, Swagata Banik

**Affiliations:** 1 San Francisco Department of Public Health, 25 Van Ness Ave., Suite 500, San Francisco, CA 94102 USA; 2 Blue Diamond Society, Dhumbarahi Height, Kathmandu, Nepal; 3 Baldwin Wallace University, 275 Eastland Rd., Berea, OH 44017 USA

**Keywords:** Stigma, HIV prevalence, Intersectionality, Lived experience, Transgender persons, Trans women, Nepal, Community co-investigator

## Abstract

**Background:**

Transgender women (trans women) in Nepal are underserved in the HIV response. Data are needed to determine the HIV prevalence disaggregated from other key populations and to identify the particular risks faced by this community. Trans women are marginalized around the world and research is also needed to determine the impact of stigma on HIV risk to inform trans-specific interventions.

**Methods:**

In 2019, we conducted the first population-based HIV behavioral surveillance study of trans women disaggregated from other key populations using respondent driven sampling (RDS). We estimated the HIV prevalence for trans women, and bivariate and multivariate Poisson binomial regression models were constructed to examine the relationship between HIV risk and stigma.

**Results:**

Trans women participants (*N* = 200) had a mean age of 33 years old (SD = 10.96). We found a weighted HIV prevalence of 11.3% (95% CI 6·82% - 18·13). We found that depression and anxiety (aPR 0.81; 95% CI 0.67–0.97; *p* = 0·02) and current engagement in sex work (aPR 1.31; 95% CI 1.01–1.71; *p* = 0·046) were significantly associated with greater prevalence of condomless receptive anal intercourse. We found that experienced stigma of ever being verbally abused due to gender identity was significantly associated with lower prevalence of depression and anxiety (aPR 0.42; 95% CI 0.20–0.87; *p* = 0·002). Feeling unaccepted in Nepali society and believing people thought they were a criminal because of their trans identity was significantly associated greater prevalence of current sex work (aPR 1.36; 95% CI 1.03–1.78; *p* = 0·03; aPR 1.45; 95% CI 1.03–2.07; *p* = 0.04). Every measure of experienced stigma assessed was significantly associated with greater prevalence of current engagement in sex work.

**Conclusions:**

Trans women are highly stigmatized in Nepal, leading to individual and systems factors that impact their risk for HIV. Interventions are needed that support the economic and mental wellbeing of trans women to prevent their heighted risk of HIV from stigma.

## Background

Transgender women (trans women) are one of the most severely impacted and underserved key populations in the global response to HIV (human immunodeficiency virus). Data from low- and middle- income countries (LMICs) finds that trans women have 37 times greater odds of having HIV than non-trans men and 77.5 times greater odds compared to non-trans women [1]. Nepal is a country in South Asia with a concentrated HIV epidemic among key populations, including trans women [2]. A recent behavioral surveillance study found a 8.2% HIV prevalence among men who have sex with men (MSM) and trans women in Nepal [3], while the general adult population prevalence was 0.15% [4]. Studies in Nepal combine trans women with MSM, but no study has estimated the prevalence of HIV among trans women alone. Aggregation of trans women with other populations likely under-estimates the effect of HIV on trans women in Nepal. More importantly, aggregated data limits what we know about the specific risks and vulnerabilities trans women in Nepal face that are different from MSM. Data are needed in Nepal to determine the scope and impact of HIV on trans women in Nepal.

Data are also needed on the drivers of HIV risk among trans women. Research outside Nepal links anti-trans stigma to family rejection, limitations in educational opportunities, unemployment, housing instability, lack of health care access, and targeted violence from law enforcement and others [5–7]. While trans women in Nepal are constitutionally recognized as a gender minority group, they face extreme discrimination, human rights violations and are socially isolated, all of which may increase their risk for HIV [8]. Our prior qualitative research found that anti-trans stigma was an important factor in the marginalization of trans women that may explain their elevated risk for HIV [8]. Anti-trans stigma is linked to adoption of the meti role (i.e., receptive sex partner) [7, 9], which is highly stigmatized in Nepal [9]. Trans women also face family rejection for not fulfilling their obligation for procreation as many do not want to get married to a cisgender woman and have children with that person [7]. Trans women in Nepal also face violence that may result in mental distress and risk behavior to cope [8]. Much like other trans women around the globe, trans women in Nepal also face employment discrimination [7].

The combined effects of external forms of stigma have known effects on social determinants of health and wellbeing for trans people. For example, employment discrimination results in impoverishment among trans women and prevents them from fulfilling their obligations for income and remittances to family [7, 10]. Lack of employment opportunities often results in exchanging sex for income wherein trans women experience stigma related to sex work and perceived HIV status [11]. Anti-trans stigma has also created high vulnerability to physical and sexual assault trans women face by clients and law enforcement officers [8]. Trans women also face internalized forms of stigma wherein they apply negative beliefs about transgender people to themselves [12]. Among trans women, internalized anti-trans stigma affects trans women’s self-esteem and has been associated with depression and trauma [13], as well as elevated suicidality [14]. In studies with trans women, internalized anti-trans stigma and discrimination is also associated with mental health issues that result in poor coping strategies, including high risk sex and substance use [15, 16]. Stigma that impacts trans women economically results in the need to do sex work, which then increases trans women’s risk of exposure to HIV from having many sexual partners and less power to negotiate condom use due to fear of violence and need for income.

The goal of this analysis was to determine, for the first time in Nepal, the HIV prevalence for trans women separate from other key populations, and to examine the relationship between anti-trans stigma and HIV risk. To establish a benchmark of risk, we first obtained a population-based respondent driven sampling (RDS) HIV point estimate for trans women. We also measured engagement in HIV-related sexual risk behavior and drivers of risk including different dimensions of anti-trans stigma. Our hypothesis was that HIV related-risk behavior would be associated with experienced and anticipated experiences of anti-trans stigma and the internalization of stigma among trans women in Nepal. The goal of this paper was to gain insight on how anti-trans stigma most impacted risk behavior to identify future intervention targets.

## Methods

### Data collection

Our study, *Sweekar* (translates as “acceptance”), was a population-based bio-behavioral study conducted in collaboration with our community research partners at the Blue Diamond Society (BDS). Together we collected a broad range of demographic, psychosocial, risk behavior and HIV testing data from 200 trans women between May and October 2019. Eligible participants were 18 years old or older, identified as transgender, hijra, Meti, third gender (assigned male sex at birth but legally recognized as “other”) category) or anything other than the gender typically associated with their male sex at birth, lived in the Kathmandu Valley and spoke Nepali and/or English. The survey was translated from English into Nepali and most survey interviews were conducted in the native tongue of Nepali. The over-arching goals for recruitment were to achieve a diverse and externally valid sample of trans women, and to innovate methods to maximize recruitment with this hidden population and therefore enhance internal validity. We began the study with three “seeds” who were recruited purposively [17] with diversity to reflect different socio-economic statuses or castes, groups within the local trans woman population, language groups, ages, educational backgrounds and identity. Seeds were recruited from key informant referrals and through contacts from our community Co-Investigator who runs BDS, which is the only lesbian, gay, bisexual and transgender (LGBT)-serving organization in Nepal. Trans women interested in being seeds came to the BDS offices to complete the survey. Trained staff who self-identified as trans or were part of the LGBT community recruited, surveyed and HIV tested participants and provided referrals for services and follow up. Seeds were asked to recruit three members of their network who were trans women in order to reduce bias from any particular individual’s network. Seeds and subsequent recruiters received an incentive for participation in the survey and HIV testing and received remuneration for all referrals who enrolled in the study. Incentives for survey participation and taking a HIV test were 1000 Nepalese rupees (NPR) (approximately $10 United States Dollar at the time of the study), and each recruit resulted in a remuneration to the participant of 500 NPR.

### Measures

Measures were developed in close collaboration between the United States (US) and Nepal-based teams with incorporation of items to meet the study aims, prior measures used in behavioral surveys in Nepal by BDS and the US team, and with input from community stakeholders. Twenty stakeholders were engaged from non-governmental organizations interested in addressing stigma in health and HIV prevention and care, researchers and academicians and HIV care providers interested in similar topics, government and civic officials, along with business leaders and the cultural community. Leaders from the trans women community were invited and their input was centered in measure development.

Demographic factors were captured to descriptively characterize trans women and their social economic status in the Nepali context. Trans women were asked about their age, gender identity, sexual orientation, birthplace, living situation, education, income, and caste. For caste we asked about the most prevalent castes and included indigenous caste, which represents the Newari people who are native to the Kathmandu Valley. We also asked about employment and marriage status. For employment, trans women were asked their level of employment (e.g., full or part time) for any type of job, including sex work, and they were asked if they were students or retired. We asked about legal marriage status as social marriage between trans women and their partners is not recognized in Nepali law. Rapid HIV testing was conducted using a serial testing scheme based on the Nepal national algorithm and using Alere Determine™ rapid HIV test kits. All participants received posttest counseling, with specific messages tailored to their test result. Persons with any reactive result, or indeterminate result, were given a referral to HIV care services at the local HIV hospital. BDS had onsite HIV testing facilities, trained staff and resources available. Sex work was measured as having exchanged sex for money, goods or a place to stay. We asked whether trans women ever engaged in sex work or were currently engaged in sex work. We also asked about the number of sexual partners each participant had in the last 6 months. We assessed whether trans women ever had receptive anal sex, with or without condoms. We asked about whether they injected drugs, binge drank, or used drugs before sex in the last year. Finally, we enumerated the participants who reported having diagnoses of both depression and anxiety in the last 12 months.

We measured three types of anti-trans stigma that we hypothesized would impact HIV risk either via discrimination leading to direct impact on condomless receptive anal intercourse, and indirectly on risk via sex work and mental distress. Building on the Health Stigma and Discrimination Framework (HSDF) [18] we used measures that represent three dimensions of stigma including internalized stigma, anticipated stigma and experienced stigma. The HSDF accounts for the intersectional nature of stigma giving us a framework to examine intersectional trans stigma to help identify areas for intervention to disrupt the pathway from stigma to risk. To do so, we have provided descriptive data on three types of stigma (internalized, anticipated and experienced) and examined the relationship with risk factors for HIV. Internalized measures of stigma we used were conforming to cisgender norms and hiding gender identity from family. Conforming to cisgender norms was measured with a “yes” response to a question asking if passing as a woman was important to the respondent’s self-esteem. Family is the bedrock and social safety net of Nepali society, [19] therefore we posit that hiding trans identity from family is an important cultural construct reflecting internalized stigma. Drawing from our formative data, we assessed anticipated stigma with two items measuring whether a participant anticipated that Nepali society was not accepting of them as a trans person and whether the participant anticipated that people in Nepal believed they were a criminal because they were trans. Specifically, we asked, “Are trans women an accepted part of Nepali society?”, and “Do people in Nepal think you are a criminal because you are trans?” Experienced stigma was measured as overt experiences of employment discrimination, arrest, and verbal, physical, or sexual abuse because they were trans. Specifically we asked, “Have you been denied employment because of your gender identity?,” “In your lifetime, do you believe you have ever been unfairly arrested for being trans?,” “Have you ever been verbally abused or harassed because of your gender identity/presentation?,” “Have you ever been physically abused or harassed because of your gender identity/presentation?,” and “Have you ever experienced sexual violence because of your gender identity/presentation?”

### Data analysis

We used RDS analysis Tool 7.1 (RDSAT; Cornell, NY) to compute individualized RDS weights accounting for the size of each participant’s network of trans women. These weights were exported from RDSAT and merged into a Stata dataset comprising participant’s demographic and behavioral data. RDS weights were used to estimate the population prevalence and corresponding 95% confidence intervals (95%CI) of various descriptive findings, including demographic characteristics, HIV-related risk behaviors, drug risk behaviors, self-reported mental health diagnoses, and stigma experiences (Tables 1, 2 and 3). Multivariable Poisson binomial regression models were constructed to examine the independent relationships between exposure to each of the three forms of stigma and (1) engagement in condomless receptive anal sex, (2) self-reported diagnoses of both depression or anxiety, or (3) (current engagement in sex work. These models adjusted for factors hypothesized to confound the exposure-outcome relationship, including participant age, birthplace, educational attainment, monthly income, and caste. Statistical significance was reached for a *p*-value less than 0.05.

**Table 1:** Crude and RDS-weighted prevalence of demographic characteristics among trans women in Nepal, 2019 (*n* = 200)

	N	Crude %	RDS-weighted %, (95% CI)
*Demographic characteristics*			
Age group (years)			
18–24	55	27.50	27.45 (19.40–37.30)
25–34	60	30.00	25.35 (17.73–34.85)
35 or older	85	42.50	47.20 (37.37–57.25)
Gender identity			
Trans woman	129	64.50	71.17 (61.23–79.42)
Other/Tesoro Lingi	71	35.50	28.83 (20.58–38.77)
Sexual orientation			
Straight or heterosexual	189	94.50	94.05 (88.46–97.02)
Gay	8	4.00	4.25 (1.82–9.63)
Other	3	1.50	1.70 (0.53–5.34)
Birthplace			
From Kathmandu	120	60.00	59.59 (51.45–67.24)
Rural-urban migrant	78	39.00	38.90 (26.21–45.59)
Living situation			
Own a house	39	19.50	21.65 (14.27–31.44)
Rent a house/room	156	78.00	73.73 (63.65–81.82)
Live with parents	5	2.50	4.61 (1.74–11.65)
Educational attainment			
No formal education	27	13.50	14.63 (8.61–23.77)
Grade school	103	51.50	53.60 (43.55–63.38)
More than grade school	70	35.00	31.77 (23.32–41.60)
Monthly income			
Less than 8000 NRS	16	8.00	2.62 (0.80–8.22)
8000–12,000 NRS	60	30.00	13.64 (8.26–21.70)
12,000–16,000 NRS	25	12.50	11.10 (6.29–18.87)
16,000–20,000 NRS	51	25.50	41.97 (32.41–52.16)
Over 20,000 NRS	37	18.50	27.29 (19.29–37.09)
Caste			
Brahmin	12	6.00	3.93 (1.56–9.54)
Chettri	39	19.50	21.80 (14.42–31.56)
Indigenous	138	69.00	66.95 (56.77–75.77)
Dalit	11	5.50	7.32 (3.45–14.87)
Current employment, including sex work			
Full-time	143	71.50	61.93 (51.61–71.28)
Part-time	25	12.50	17.91 (11.51–26.79)
Student	13	6.50	5.58 (2.24–13.24)
Unemployed	12	6.00	11.51 (6.15–20.50)
Retired	5	2.50	3.07 (0.96–9.36)
Legal marriage status			
Single	135	67.50	62.55 (52.21–71.87)
Married	58	29.00	32.35 (23.48–42.69)
Divorced or separated	7	3.50	5.10 (2.09–11.92)

*CI* Confidence interval

**Table 2:** Crude and RDS-weighted prevalence of HIV-related sexual risk behaviors, drug risk behaviors, and mental health diagnoses among trans women in Nepal, 2019 (*n* = 200)

	N	Crude %	RDS-weighted %, (95% CI)
*HIV-related sexual risk behaviors*			
Currently engaged in sex work	120	60.00	57.30 (47.05–66.95)
Number of sexual partners, last 6 months			
0	7	3.50	1.38 (0.38–4.79)
1 to 2	28	14.00	19.43 (12.32–29.27)
3 to 5	12	6.00	6.53 (2.73–14.82)
6+	153	76.50	72.67 (62.33–81.03)
Condomless receptive anal sex, last 6 months	154	77.00	82.64 (73.36–89.17)
*Drug risk behaviors*			
Ever injected drugs	3	1.50	1.60 (0.27–9.08)
Binge drank, last 12 months	158	79.00	75.82 (66.09–83.45)
Drug use before sex, last 12 months	51	25.50	24.25 (17.22–32.99)
*Self-reported mental health diagnoses*			
Depression and anxiety	89	44.50	26.85 (18.80–36.79)

*CI* Confidence interval

**Table 3:** Crude and RDS-weighted prevalence of internalized, anticipated, and experienced anti-trans stigma experienced by trans women in Nepal, 2019 (*n* = 200)

	N	Crude %	RDS-weighted %, (95% CI)
*Internalized stigma*			
Conformity to cisgender norms	91	45.50	38.93 (29.83–48.86)
Hiding of gender identity from family	178	89.00	87.86 (79.32–93.17)
*Anticipated stigma*			
Belief that trans women are not accepted in Nepali society	133	66.50	71.20 (61.25–79.45)
Belief that people think the participant is a criminal because they are trans	152	76.00	72.32 (62.22–80.56)
*Experienced stigma*			
Ever denied employment for being trans	138	69.00	78.62 (68.93–85.91)
Ever arrested for being trans	104	52.00	55.34 (45.14–65.11)
Verbally abused for being trans	191	95.50	95.07 (87.20–98.20)
Physically abused for being trans	181	90.50	86.36 (76.80–92.38)
Sexually abused for being trans	168	84.00	77.37 (66.99–85.21)

*CI* Confidence interval

Our study received human subject approval from the University of California, San Francisco, Baldwin Wallace University and the Nepal Health Research Council.

## Results

Trans women recruited for our study (*N* = 200) were between the ages 18 to 67 years old, with a mean age of 33 years old (SD = 10.96). Almost half of trans women in Nepal were 35 years of age or older (Table 1). Almost all were straight/heterosexual. More than half migrated to Kathmandu. Most trans women rented a house/room rather than live with their family. Many trans women had no formal education (14.63, 95% CI 8.61–23.77) and about half had 5-years of a grade school education. Most had a full-time job, which included those who did sex work for a living. Over half, identified as indigenous, meaning they were from the Kathmandu Valley. Most were currently single. We found a weighted HIV prevalence of 11.3% (95% CI 6.82% - 18.13).

Table 2 presents RDS-weighted drivers of HIV risk, including sexual and drug use behaviors and mental health. About one third had at least one sexually transmitted disease (STD) in the past 12 months. A total of 57.30% reported being a sex worker currently (95% CI 47.05–66.95). Over 72% had six or more sexual partners (95% CI 62.33–81.03) and 82.64% reported having condomless receptive anal sex in the last 6 months (95% CI 73·36–89.17). Only 1.60% had ever used injection drugs (95% CI 0.27–9.08) while 75.82% had binge drank at least once in the last year (95% CI 66.09–83.45). Almost 27% (95% CI 18.80–36.79) reported a diagnosis of both depression and anxiety.

Experiences of stigma and discrimination were overall high in our sample (Table 3). About 38.93% felt passing was important to their self-esteem (95% CI 29.83–48.86). For another measure of internalized stigma, we found that most (87.86 95% CI 79.32–3.17) hid their gender identity from family. Experiences of anticipated stigma were also high. Over two thirds, or 71.20% (95% CI 61.25–79.45) of our participants felt unaccepted in Nepali society and 72.32% (95% CI 62.22–80.56) believed that Nepali people thought trans women were criminals because of their gender identity. Experienced stigma experiences were also high. Over 78% participants had been denied employment for being trans (95% CI 68.93–85.91). About half our participants reported being arrested for being trans (55.34%; 95% CI 45.14–65.11). The majority of participants experienced verbal (95.07%; 95% CI 87.20–98.20), physical (86.36%; 95% CI 76.80–92.38), and sexual (77.37%; 95% CI 66.99–85.21) abuse.

After adjusting for RDS-weights and covariates in the multivariable Poisson binomial regression model, we found that current engagement in sex work (adjusted prevalence ratio, aPR 1.31; 95% CI 1.01–1.71; *p* = 0·046) was significantly associated with greater prevalence of condomless receptive anal intercourse (Table 4). Reporting diagnoses of both depression and anxiety were significantly associated with lower prevalence of condomless receptive anal intercourse (aPR 0.81; 95% CI 0.67–0.97; *p* = 0.02).

**Table 4:** RDS-weighted associations between anti-trans stigma and condomless receptive anal intercourse among trans women in Nepal, 2019 (*n* = 200)

	Condomless receptive anal sex, last 6 months		
	aPR	(95% CI)	***p***-value
*Characteristics*			
Self-reported depression and anxiety	0.81	(0.67–0.97)	0.02
Sex Work	1.31	(1.01–1.71)	0.046
*Internalized stigma*			
Conformity to cisgender norms	1.17	(0.99–1.39)	0.06
Hiding of gender identity from family	0.98	(0.81–1.18)	0.83
*Anticipated stigma*			
Belief that trans women are not accepted in Nepali society	1.25	(0.97–1.62)	0.09
Belief that people think the participant is a criminal because they are trans	1.06	(0.83–1.34)	0.65
*Experienced stigma*			
Ever denied employment for being trans	1.02	(0.78–1.33)	0.89
Ever arrested for being trans	1.09	(0.91–1.31)	0.34
Verbally abused for being trans	1.89	(0.58–6.11)	0.29
Physically abused for being trans	1.29	(0.81–2.09)	0.28
Sexually abused for being trans	1.10	(0.83–1.47)	0.49

*aPR* Prevalence ratio adjusting for participant age, birthplace, educational attainment, monthly income, and caste, *CI* Confidence interval

Participants who experienced verbal abuse due to gender identity were significantly less likely to experience both depression and anxiety (aPR 0.42; 95% CI 0.20–0.87; *p* = 0·02) (Table 5). The belief that trans women are not accepted in Nepali society (aPR 1.36; 95% CI 1.03–1.78; *p* = 0·03) and the belief that others think the participant is a criminal due to their gender identity (aPR 1.45; 95% CI 1.02–2.07; *p* = 0·04) were both significantly associated with greater adjusted prevalence of engaging in sex work. Also, ever being denied employment for being trans (aPR 1.94; 95% CI 1.11–3.37; *p* = 0.02), ever being arrested due to gender identity (aPR 2.35; 95% CI 1.58–3.51; *p* <  0.01), and ever being verbally (aPR 140.17; 95% CI 13.61–1443.97 *p* <  0.01), physically (aPR 8.62; 95% CI 1.53–48.70; *p* = 0.02), or sexually (aPR 2.65; 95% CI 1.22–5.74; *p* = 0.01) abused due to gender identity were significantly associated with greater likelihood of engaging in sex work.

**Table 5:** RDS-weighted associations between anti-trans stigma and (1) self-reported diagnoses of both depression and anxiety, or (2) current sex work among trans women in Nepal, 2019 (*n* = 200)

	Self-reported diagnoses of both depression and anxiety			Current sex work		
	aPR	(95% CI)	***p***-value	aPR	(95% CI)	***p***-value
*Internalized stigma*						
Conformity to cisgender norms	1.04	(0.57–1.90)	0.89	0.96	(0.74–1.25)	0.78
Hiding of gender identity from family	1.11	(0.49–2.52)	0.79	1.26	(0.93–1.69)	0.14
*Anticipated stigma*						
Belief that trans women are not accepted in Nepali society	0.87	(0.43–1.77)	0.70	1.36	(1.03–1.78)	0.03
Belief that people think the participant is a criminal because they are trans	1.48	(0.62–3.54)	0.38	1.45	(1.02–2.07)	0.04
*Experienced stigma*						
Ever denied employment for being trans	1.84	(0.90–3.76)	0.09	1.94	(1.11–3.37)	0.02
Ever arrested for being trans	0.98	(0.53–1.82)	0.96	2.35	(1.58–3.51)	< 0.01
Verbally abused for being trans	0.42	(0.20–0.87)	0.02	140.17	(13.61–1443.97)	< 0.01
Physically abused for being trans	0.76	(0.33–1.76)	0.52	8.62	(1.53–48.70))	0.02
Sexually abused for being trans	0.84	(0.42–1.68)	0.63	2.65	(1.22–5.74))	0.01

*aPR* Prevalence ratio adjusting for participant age, birthplace, educational attainment, monthly income, and caste, *CI* Confidence interval

## Discussion

Our population-based HIV point estimate for trans women in Nepal of 11.3% was more than twice a recent pooled prevalence of 5% among MSM/trans women in Nepal [20]. Based on these data, HIV among trans women alone may be driving the aggregated HIV estimates for MSM/trans women in Nepal. Our data are consistent with data from trans women in the Terai highway districts finding that 13% were living with HIV [21]. The Terai highway districts is the region of the country facing the highest HIV risk due an open border with India that enables sex and drug trafficking [22–24]. Trans women in Kathmandu Valley face an environment with similar risk as the border regions.

The level of experienced stigma we found was consistent with the body of literature on anti-trans motivated violence against trans women globally. A systematic review [25] found that as many as 50% of trans people experience physical and sexual violence motivated by the perception of sexual or gender minority status. Trans women in Nepal also faced considerable internalized stigma as almost half internalized that they needed to conform to cisgender norms and the vast majority hid their gender identity from family. It was also notable that few trans women in our study lived with their families. While living within an extended family system is central to the collectivistic tradition of Nepal, less than 3% of our participants reported that they lived with their parents. As a result, many trans women in Nepal did not benefit from the most important source of social and economic support in Nepalese culture, i.e., family [19, 26].

While we did not find a direct relationship between anti-trans stigma and condomless receptive anal intercourse as our main HIV risk behavior, we did find anti-trans stigma was significantly associated with mental health distress and sex work. Mental distress is a known driver of engagement in HIV-related sexual risk behavior among trans women [27]. Much of the research on the impact of mental distress on trans women has assessed HIV risk related to syndemics of violence, substance use and mental health [28, 29], with less being known about the direct impact on externalizing HIV-related risk behaviors. Our data show that experienced stigma was significantly associated with mental distress and engagement in sex work currently, both of which are associated with elevated HIV risk among trans women [29–31].

In prior qualitative research, we found that sex work was one of very few viable employment options for trans woman in Nepal due to anti-trans stigma [8]. Trans women are also more vulnerable as sex workers than cisgender women because they have less power to negotiate condom use with their clients [8, 32]. Trans women in Nepal are also regularly harassed by police [8]. Though sex work is not explicitly criminalized in Nepal, anti-trafficking laws are used to harass and arrest sex workers [33]. In attempts to prevent arrest and harassment from law enforcement, trans women do not carry condoms while working to prevent law enforcement from having “evidence” for their arrest [8]. Trans women sex workers in Nepal are also at risk of HIV from sexual assault by law enforcement officers, perhaps in part explaining our finding that sexual abuse was significantly associated with sex work. Our data on sexual violence towards trans women is consistent with the literature finding that trans women face anti-trans violence from intimate partners and police [8, 34, 35]. Sexual abuse may have also predated trans women’s engagement in sex work and created higher risk of engagement in sex work. Longitudinal research is needed to investigate this causal relationship.

A limitation to our study was that data are cross-sectional with no temporality. Thus, our findings may not establish the directionality of the relationship between anti-trans stigma, mental health and HIV risk. STD tests for infections other than HIV were also not conducted, which may have resulted in an under-reporting of disease. The mental health indicators in our study were also self-reported and cultural meanings of different mental health conditions may have influenced self-report. Lastly, our sample may be over-represented by trans women who were lower income as we provided an incentive for participation and remuneration for successful recruits. Despite these limitations, our study is the first in Nepal, to our knowledge, that estimates the HIV prevalence among trans women disaggregated from MSM.

## Conclusions

Much like in other places around the world, we found that trans women are highly and disproportionately impacted by HIV. Our findings point to the importance of assessing HIV risk among trans women as a stand-alone key population so that their specific needs for HIV prevention and care are addressed. Trans women in Nepal faced extraordinary stigma, discrimination and violence. The negative impact of stigma on health is well supported in the literature [36] and stigma is a known contributor to health disparities, including HIV [37]. We found that trans women were stigmatized at the individual, interpersonal and community level, with the greatest and most intervenable factor being the impact of stigma on trans women’s economic circumstances. Interventions are needed to address stigma towards trans women in Nepal at multiple levels and to create economic opportunity. Such interventions can reduce the psychological and economic stress trans women face from stigma and serve as a protective factor for HIV risk.

## Data Availability

The datasets generated and/or analyzed during the current study are not publicly available due to the sensitive nature of the data and the study population, which is highly stigmatized in Nepal, but they are available from the corresponding author on reasonable request.

## Abbreviations

Trans women: Transgender women
HIV: Human immunodeficiency virus
LMICs: Low- and middle- income countries
MSM: Men who have sex with men (MSM)
RDS: Respondent driven sampling
BDS: Blue Diamond Society
NPR: Nepalese rupees
LGBT: Lesbian, gay, bisexual and transgender
US: United States
HSDF: Building on the Health Stigma and Discrimination Framework
STD: Sexually transmitted disease

## References

[1] Baral SD, Poteat T, Stromdahl S, Wirtz AL, Guadamuz TE, Beyrer C (2013). Worldwide burden of HIV in transgender women: a systematic review and meta-analysis. Lancet Infect Dis.

[2] Paudel T, Singh N, Raj Banjara M, Kafle SP, Chandra Ghimire Y, Pokharel BR (2016). Epidemiology of HIV, programmatic progress and gaps in last 10 years in Nepal. J Virus Erad.

[3] Ministry of Health and Population NCfAaSCN (2018). Integrated biological and behavioral surveillance (IBBS) survey among men who have sex with men (MSM) and transgender (TG) in Tarai highway districts of Nepal- 2018 (round –II)- factsheet.

[4] Ministry of Health and Population (2017). Annual report.

[5] Santis J (2009). HIV infection risk factors among male-to-female transgender persons: a review of the literature. J Assoc Nurses AIDS Care.

[6] Khan SI, Hussain MI, Gourab G, Parveen S, Bhuiyan MI, Sikder J (2008). Not to stigmatize but to humanize sexual lives of the transgender (hijra) in Bangladesh: condom chat in the AIDS era. J LGBT Health Res.

[7] Wilson E, Pant SB (2010). Stigma and HIV risk behavior of transgender women in Nepal: implications for HIV prevention. Retrovirology.

[8] Wilson E, Pant SB, Comfort M, Ekstrand M (2011). Stigma and HIV risk among Metis in Nepal. Cult Health Sex.

[9] Deuba K, Karki DK, Shrestha R, Aryal UR, Bhatta L, Rai KK (2014). Risk of HIV infection among men having sex with men in Kathmandu Valley, Nepal. Asia Pac J Public Health.

[10] Wilson EC, Garofalo R, Harris RD, Herrick A, Martinez M, Martinez J (2009). Transgender female youth and sex work: HIV risk and a comparison of life factors related to engagement in sex work. AIDS Behav.

[11] Bao A, Colby DJ, Trang T, Le BQ, Dinh TD, Nguyen QH (2016). Correlates of HIV testing among transgender women in Ho Chi Minh, Vietnam. AIDS Behav.

[12] Earnshaw VA, Chaudoir SR (2009). From conceptualizing to measuring HIV stigma: a review of HIV stigma mechanism measures. AIDS Behav.

[13] Lacombe-Duncan A, Warren L, Kay ES, Persad Y, Soor J, Kia H, et al. Mental health among transgender women living with HIV in Canada: findings from a national community-based research study. AIDS Care. 2020:1–9. 10.1080/09540121.2020.1737640. 10.1080/09540121.2020.1737640 32172609

[14] Perez-Brumer A, Hatzenbuehler ML, Oldenburg CE, Bockting W (2015). Individual- and structural-level risk factors for suicide attempts among transgender adults. Behav Med.

[15] Wilson EC, Chen YH, Arayasirikul S, Raymond HF, McFarland W (2016). The impact of discrimination on the mental health of trans*female youth and the protective effect of parental support. AIDS Behav.

[16] Turner CM, Santos GM, Arayasirikul S, Wilson EC (2017). Brief report: psychosocial predictors of engagement in sexual risk behavior among trans*female youth aged 16-24 years in San Francisco. J Acquir Immune Defic Syndr.

[17] Magnani R, Sabin K, Saidel T, Heckathorn D (2005). Review of sampling hard-to-reach and hidden populations for HIV surveillance. AIDS.

[18] Stangl AL, Earnshaw VA, Logie CH, van Brakel W, Simbayi LC, Barre I (2019). The Health Stigma and Discrimination Framework: a global, crosscutting framework to inform research, intervention development, and policy on health-related stigmas. BMC Med.

[19] Skordis J, Pace N, Vera-Hernandez M, Rasul I, Fitzsimons E, Osrin D (2019). Family networks and healthy behaviour: evidence from Nepal. Health Econ Policy Law.

[20] Deuba K, Sapkota D, Shrestha U, Shrestha R, Rawal BB, Badal K (2020). Effectiveness of interventions for changing HIV related risk behaviours among key populations in low-income setting: a meta-analysis, 2001-2016. Sci Rep.

[21] Storm M, Deuba K, Damas J, Shrestha U, Rawal B, Bhattarai R, et al. Prevalence of HIV, syphilis, and assessment of the social and structural determinants of sexual risk behaviour and health service utilisation among MSM and transgender women in Terai highway districts of Nepal: findings based on an integrated biological and behavioural surveillance survey using respondent driven sampling. 2019. 10.1186/s12879-020-05122-3 PMC7282139 32513134

[22] Deuba K, Anderson S, Ekstrom AM, Pandey SR, Shrestha R, Karki DK (2016). Micro-level social and structural factors act synergistically to increase HIV risk among Nepalese female sex workers. Int J Infect Dis.

[23] Deuba K, Ekstrom AM, Tomson G, Shrestha R, Marrone G (2017). HIV decline associated with changes in risk behaviours among young key populations in Nepal: analysis of population-based HIV prevalence surveys between 2001 and 2012. Int J STD AIDS.

[24] Cousins S (2018). Blue diamond society: working with Nepal’s LGBT community. Lancet HIV.

[25] Blondeel K, de Vasconcelos S, Garcia-Moreno C, Stephenson R, Temmerman M, Toskin I (2018). Violence motivated by perception of sexual orientation and gender identity: a systematic review. Bull World Health Organ.

[26] Wali N, Renzaho AMN (2018). “Our riches are our family”, the changing family dynamics & social capital for new migrant families in Australia. PLoS One.

[27] Clements-Nolle K, Marx R, Guzman R, Katz M (2001). HIV prevalence, risk behaviors, health care use, and mental health status of transgender persons: implications for public health intervention. Am J Public Health.

[28] Poteat T, Scheim A, Xavier J, Reisner S, Baral S (2016). Global epidemiology of HIV infection and related syndemics affecting transgender people. J Acquir Immune Defic Syndr.

[29] Parsons JT, Antebi-Gruszka N, Millar BM, Cain D, Gurung S (2018). Syndemic conditions, HIV transmission risk behavior, and transactional sex among transgender women. AIDS Behav.

[30] Seekaew P, Pengnonyang S, Jantarapakde J, Sungsing T, Rodbumrung P, Trachunthong D (2018). Characteristics and HIV epidemiologic profiles of men who have sex with men and transgender women in key population-led test and treat cohorts in Thailand. PLoS One.

[31] Becasen JS, Denard CL, Mullins MM, Higa DH, Sipe TA. Estimating the prevalence of HIV and sexual behaviors among the US transgender population: a systematic review and meta-analysis, 2006-2017. Am J Public Health. 2019;109(1):e1–e8. 10.2105/AJPH.2018.304727. Epub 2018 Nov 29. PMID: 30496000; PMCID: PMC6301428. 10.2105/AJPH.2018.304727 PMC6301428 30496000

[32] Ghimire L, Smith WC, van Teijlingen ER (2011). Utilisation of sexual health services by female sex workers in Nepal. BMC Health Serv Res.

[33] Ghimire L, Smith WC, van Teijlingen ER, Dahal R, Luitel NP (2011). Reasons for non- use of condoms and self- efficacy among female sex workers: a qualitative study in Nepal. BMC Womens Health.

[34] Garthe RC, Hidalgo MA, Hereth J, Garofalo R, Reisner SL, Mimiaga MJ (2018). Prevalence and risk correlates of intimate partner violence among a multisite cohort of young transgender women. LGBT Health.

[35] Gamarel KE, Reisner SL, Laurenceau JP, Nemoto T, Operario D (2014). Gender minority stress, mental health, and relationship quality: a dyadic investigation of transgender women and their cisgender male partners. J Fam Psychol.

[36] Richman LS, Hatzenbuehler ML (2014). A multilevel analysis of stigma and health: implications for research and policy. Policy Insights Behav Brain Sci.

[37] Hatzenbuehler ML, Phelan JC, Link BG (2013). Stigma as a fundamental cause of population health inequalities. Am J Public Health.

